# No Coil® placement in patients undergoing left hemicolectomy and low anterior resection for colorectal cancer

**DOI:** 10.1186/s12957-020-02096-z

**Published:** 2020-12-10

**Authors:** Michele Ammendola, Michele Ruggiero, Carlo Talarico, Riccardo Memeo, Giorgio Ammerata, Antonella Capomolla, Rosalinda Filippo, Roberto Romano, Socrate Pallio, Giuseppe Navarra, Severino Montemurro, Giuseppe Currò

**Affiliations:** 1 Science of Health Department, Digestive Surgery Unit, “Mater Domini” Hospital, University “Magna Graecia” Medical School, Viale Europa, Germaneto, 88100 Catanzaro, Italy; 2 Hepato-Biliary and Pancreatic Surgical Unit, “F. Miulli” Hospital, Acquaviva delle Fonti, Bari, Italy; 3 Department of Clinical and Experimental Medicine, Digestive Diseases Endoscopy Unit, “G. Martino” Hospital, University of Messina, Messina, Italy; 4 Department of Human Pathology of Adult and Evolutive Age, Surgical Oncology Division, “G. Martino” Hospital, University of Messina, Messina, Italy

**Keywords:** No coil, Postoperative ileus, Anastomotic leak, Left hemicolectomy, Anterior resection, Colorectal cancer, Endorectal tube

## Abstract

**Background:**

Colorectal cancer (CRC) is the most common tumor of the gastrointestinal tract. Anastomotic leak (AL) and prolonged postoperative ileus (PPOI) are two important complications of colorectal surgery. In this observational retrospective study, we evaluated the positive effects of transanal tube No Coil**®** in patients with CRC undergoing low anterior resection (LAR) and left hemicolectomy (LC).

**Methods:**

Thirty-eight cases and forty controls resulted eligible for the final sample. No Coil**®** placement (SapiMed Spa, Alessandria, Italy) was considered an inclusion criteria for the case group. No Coil**®** was placed immediately after the end of surgical treatment.

**Results:**

PPOI was significantly more frequent in the control group. AL was evident in 1 patient (2.6%) of cases and 3 patients (7.5%) of controls. No statistical difference was found in AL occurrence between groups. POI days and AL resulted associated with hospital stay. POI days were negatively associated with No Coil placement and positively with AL.

**Conclusion:**

With our preliminary data, we suggest that No Coil**®** placement can be considered as a valuable procedure assisting colorectal surgery, but further studies are required to confirm and enlarge actual evidence.

## Introduction

Colorectal cancer (CRC) is the most frequent tumor of the gastrointestinal tract, and its predicted prevalence is estimated to rise up to 2.5 million in 2035 [1, 2].

During 2018, 704.000 new cases of rectal cancer (RC) have been reported for which low anterior resection (LAR) remains the cornerstone of curative intent treatment, providing the best results in terms of quality of life [3, 4]. On the other hand, left colon cancer, which affects splenic flexure, descending colon, and sigma, is also frequent with 138,377 of new cases reported in 2014, and left hemicolectomy (LC) is the surgical technique of choice [5].

Anastomotic leak (AL), defined as a defect of the intestinal wall occurring in the anastomotic site, leads to a communication between the intra- and extraluminal compartments and is the most important complication of colorectal surgery [6–8]. There is no consensus about the prevalence of AL, since it varies depending on the site of anastomosis, with colo-colonic leak frequency being up to 0–9% and colorectal and coloanal leak rising to 20% [7–9].

Another important complication is prolonged postoperative ileus (PPOI), affecting up to 10% of patients undergoing colorectal surgery [10]. PPOI is defined as the temporary reduction or absence of gastrointestinal motility after surgery and is clinically evident with the absence of flatus and stools for at least 5 days following open abdominal surgery [11, 12]. Several factors may contribute to PPOI occurrence, and the secondary increase of intraluminal pressure is strongly associated with AL. For rectal cancer, preoperative radio-chemotherapy treatment does not represent a statistically significant risk factor; the level of anastomosis is probably the most important [13].

Defunctioning stoma is the elective procedure to prevent AL to take place after LAR [14]. Nevertheless, this procedure is burdened by several complications (e.g., longer hospital stay, reversal procedure, greater inpatient costs, permanent stoma, stoma-related complications, and patient discomfort) [14, 15]. Given the evidence that increased intraluminal rectal pressure is among the major contributors to AL [16–19], several endorectal devices (e.g., transanal tube cuff rectum, drainage tube, silicone transanal tube) have been proposed as promising alternatives to defunctioning stoma [13, 20, 21].

**Fig. 1:**
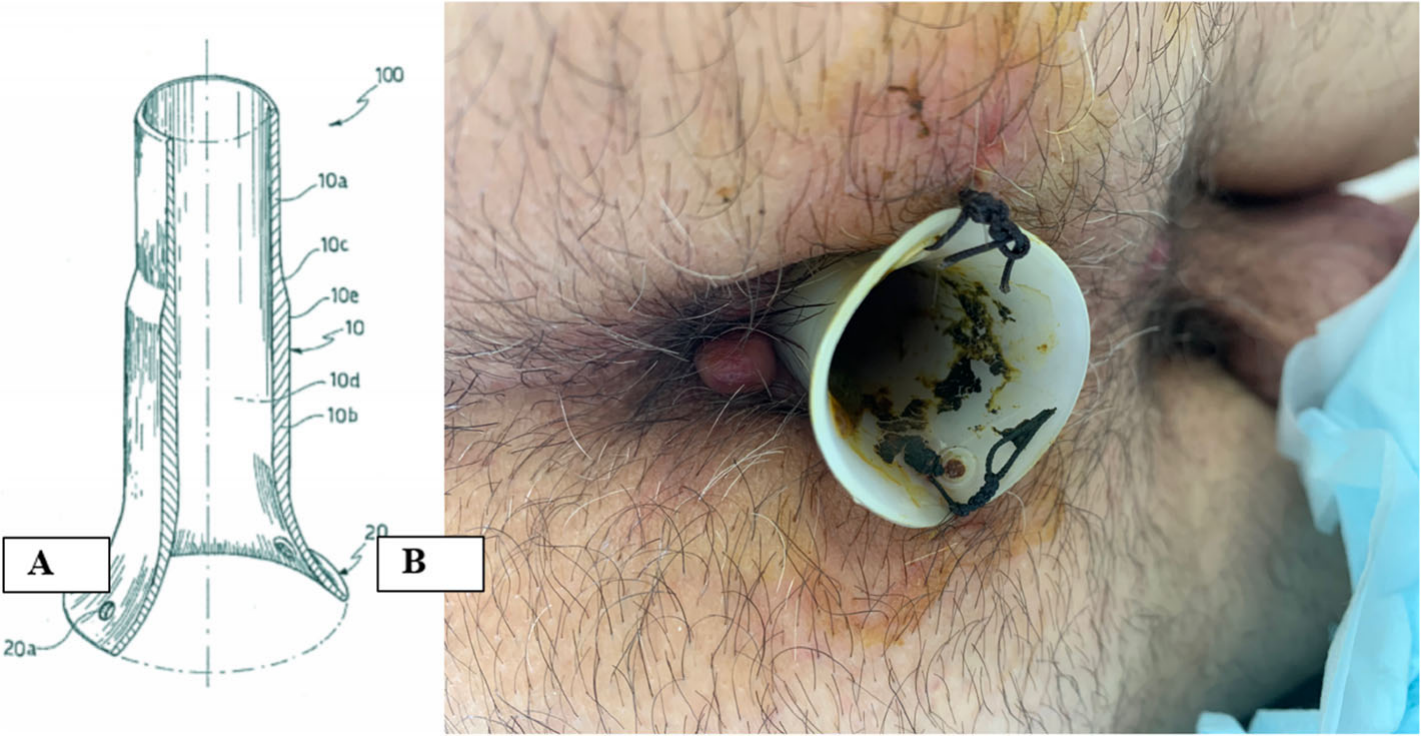
No Coil structure and the postoperative placement. **a** Length of 60–80 mm, thickness of 2 mm, and diameter of 20 mm. **b** Stabilized 6–8 cm far from the anus through two stitches

No Coil® is a transanal silicone stent that allows endorectal decompression, and it is used for anastomosis of the lower gastrointestinal tract (Fig. 1) [13].

According to recent studies, No Coil may be promising in the prevention of AL-related complications, in addition to show good feasibility, cost-effectiveness, and favorable patients’ quality of life after treatment [22]. However, evidence about No Coil implementation in the surgical treatment of CRC is limited to few studies, and definitive conclusions in terms of efficacy cannot be drawn. Moreover, these studies examined No Coil after LAR approach, and evidence about its efficacy after LC is still missing [13, 23].

Present observational retrospective study aimed at extending actual knowledge about No Coil effects in patients with CRC undergoing LAR or LC. To this extent, hospital stay, PPOI, and AL events were examined and compared between patients according to No Coil placement. No Coil use was hypothesized to reduce the occurrence of all outcomes of interest, but given the exploratory nature of the study, no definite hypothesis was postulated.

## Materials and methods

An observational retrospective case-control study was performed at the Science of Health Department, Digestive Surgery Unit, University “Magna Graecia” Medical School, “Mater Domini” Hospital of Catanzaro and examined hospital electronic medical records of patients diagnosed with CRC that underwent elective surgical intervention (LAR, LC) between January 2017 and January 2020.

Diagnosis of left colon cancer (splenic flexure, descending colon, and sigma) or rectum cancer, histological type of adenocarcinoma, T_2-4_N_0-2ab_M_0_ staging for the colon and T_2-3_N_0-2ab_M_0_ for the rectal, no preoperative radio-chemotherapy treatment, BMI range 24.0 to < 30, consecutive patients, and valid consent were considered inclusion criteria for both the case and control groups. No Coil**®** placement (SapiMed Spa, Alessandria, Italy) was considered another inclusion criteria for the case groups. Thirty-eight cases and forty controls resulted eligible for the final sample.

All participants were screened with serum carcino-ebryonic antigen (CEA) and carbohydrate antigen 19.9 (CA-19.9) blood level measurement, total body computed tomography (CT), and colonoscopy, and histopathological grading was performed according to the American Joint of Committee on Cancer (AJCC) 8^th^ Edition. Surgical approach was defined according to the European Consensus Conference [24] and The Tripartite Consensus Conference on Definitions for Anorectal Physiology and Rectal Cancer [25]. Both types of procedure (LC and LAR) were conducted in open surgery for contraindications to laparoscopy approach (comorbidity, previous surgical treatment); complete mobilization of the splenic flexure with end-to-end isoperistaltic anastomotic was performed at all participants.

No Coil silicone tube had the following characteristics: length of 60–80 mm, thickness of 2 mm, and diameter of 20 mm (Fig. 1). No Coil was placed immediately after the anastomosis, inserted through the anal sphincter, and stabilized 6–8 cm far from the anus through two stitches, then removed on the seventh postoperative day if no signs of leakage occurred (Fig. 1).

Postoperative AL and PPOI events were recorded during hospital stay and coded as 0 (no event) or 1 (event). AL was evaluated according to the Clavien–Dindo classification [26, 27]. PPOI was considered to occur when flatus and stools were absent for at least 5 days following the open abdominal surgical intervention [28, 29]. Postoperative ileus (POI) was also considered in days from the surgery to the canalization. Hospital stay was coded in days starting from the admission to the surgical department.

All procedures included in the protocol complied with the ethical standards of Helsinki Declaration and according to the Guideline for Good Clinical Practice. The Human Investigation Committee (IRB) of University “Magna Graecia” Medical School, “Mater Domini” Hospital, approved this study (Protocol N° 182, 18 June 2020).

To assess the postoperative quality of life, the patients compiled the EORTC QLQ-C30 questionnaire. A valid, informed consent for elective surgery, as well as for the collection, managing, and manipulation of medical records for scientific aims, was acquired before any further step took place.

### Statistical analysis

Statistical analyses were performed using the Statistical Package for the Social Sciences (SPSS) version 21.0 (SPSS 21.0; SPSS Inc., Chicago, IL, USA). Descriptive statistics included frequencies and percentages, and means and standard deviations, as appropriate. Differences between cases and controls were subsequently explored through *χ*^2^ for categorical variables, and *T* test for continuous variables. Stepwise linear regression was run to ascertain the association between hospital stay (dependent variable) and No Coil placement, type of surgery, POI, PPOI, and AL events (independent variables). The same procedure was applied for POI (independent variables: No Coil, type of surgery, AL). The association between AL and PPOI (dependent variables) and No Coil placement, type of intervention, and respectively POI/PPOI or AL were investigated with forward-stepwise logistic regression. Significance level was set at *p* < 0.05.

## Results

Results from the descriptive analysis and comparison between cases and controls are shown in Table 1. No differences emerged in gender and age distribution between groups. LC was performed in 24 cases (63%) and 26 controls (65%) (*χ*^2^ = 0.029; *p* = 0.526). No cases patient reported postoperative incontinence or constipation.

**Table 1:** Descriptive and comparison between groups

		Cases		Controls			
		(*N* = 38)		(*N* = 40)		χ2/t	p
Age^a^		72.6	6.5	70.8	7.4	− 1.173	0.244
Gender^b^	Male	18	47.4	22	55	0.454	0.327
	Female	20	52.6	18	45		
Type of intervention^b^	LAR	14	36.8	14	35	0.029	0.526
	LC	24	63.2	26	65		
Hospital stay^a^		12.1	4.7	16.6	6.8	3.494	0.001
POI^a^		3.8	0.9	7.9	3.5	7.193	< 0.001
PPOI^b^		9	23.7	40	100	48.593	< 0.001
AL^b^		1	2.6	3	7.5	0.949	0.327

*LC* left hemicolectomy, *LAR* low anterior resection, *POI* postoperative ileus days, *PPOI* prolonged postoperative ileus, *AL* anastomotic leak

^a^Mean and SD; ^b^ fr, %.

**Fig. 2:**
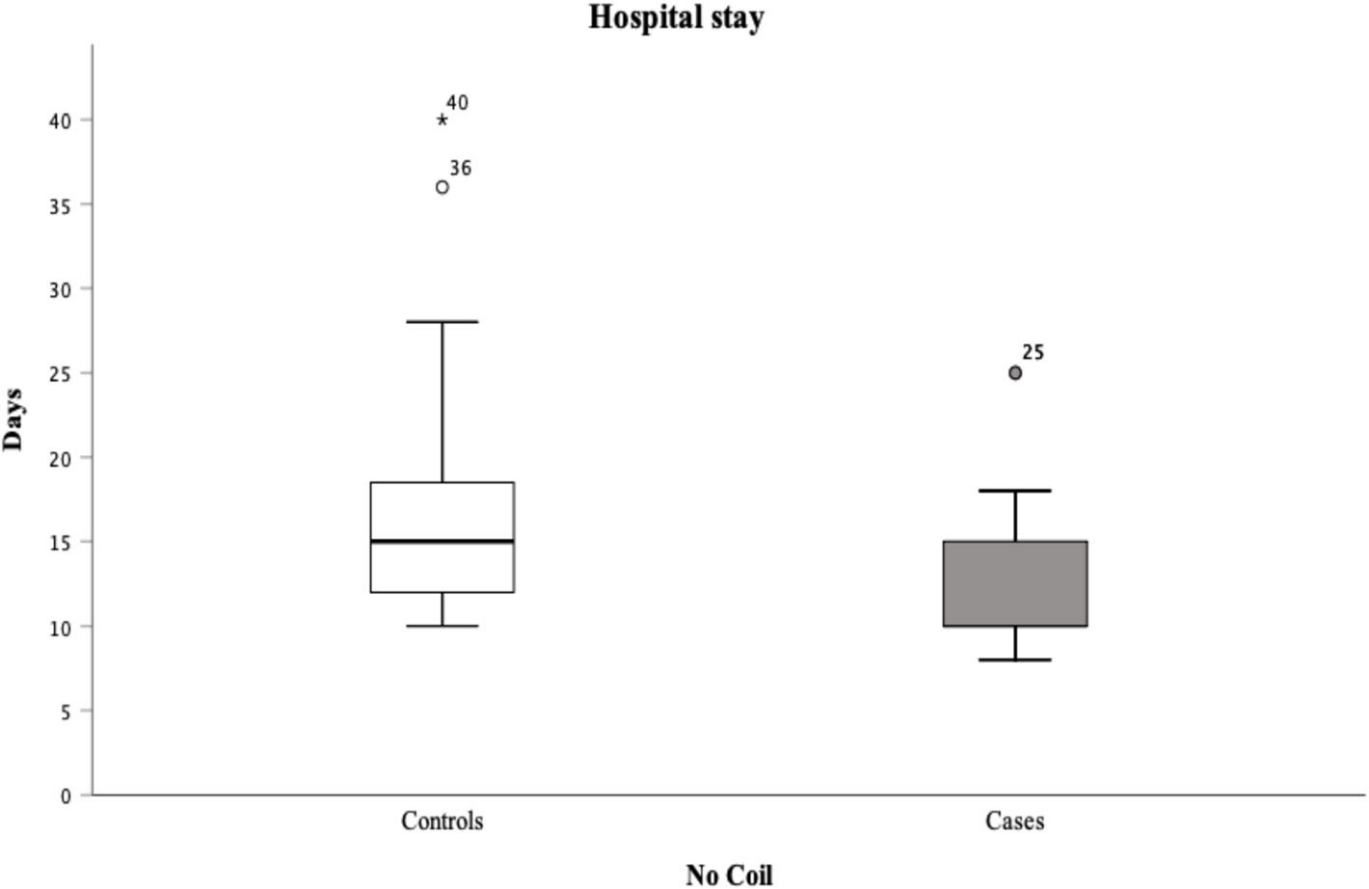
Hospital stay in cases and controls

**Fig. 3:**
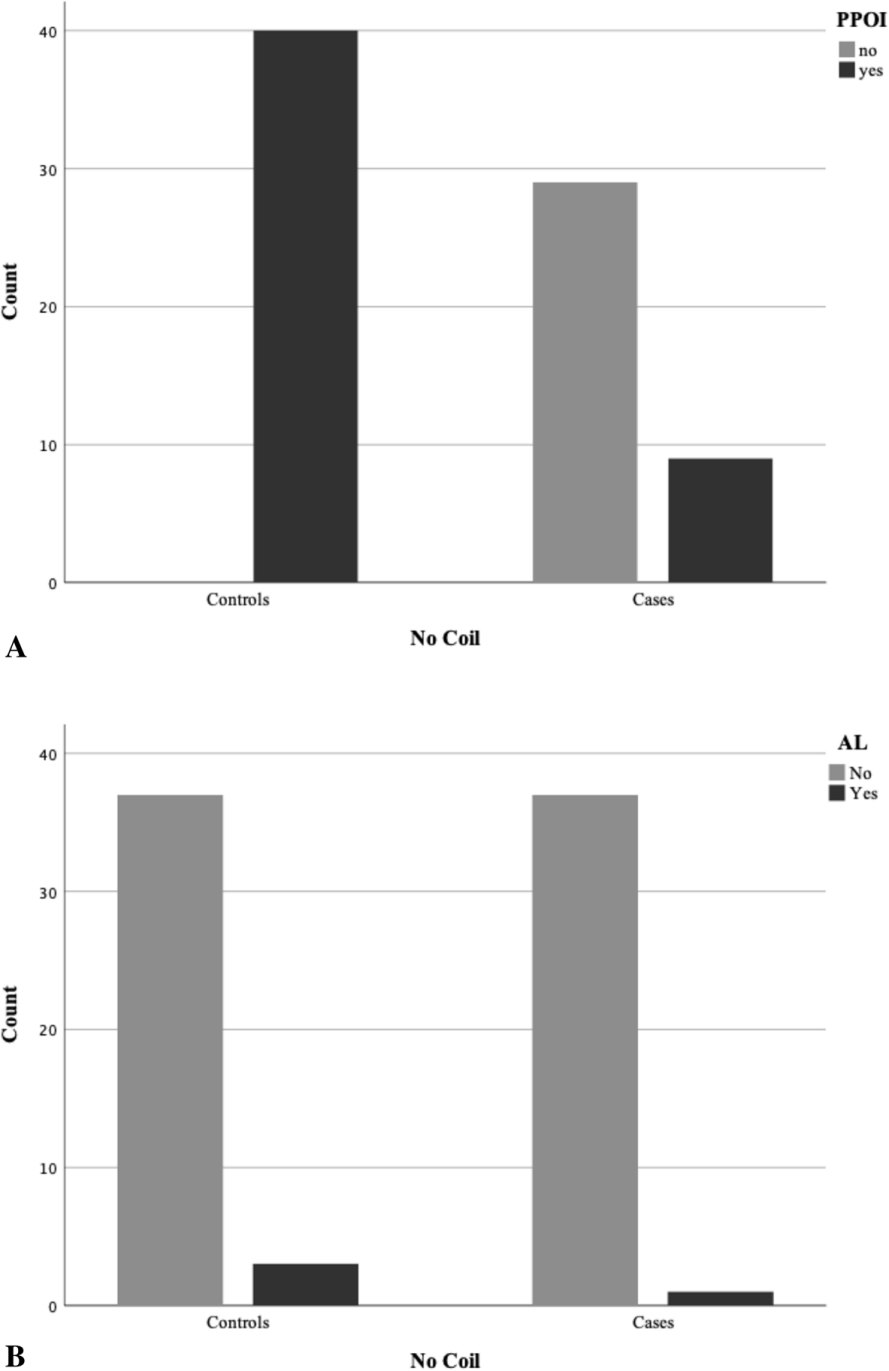
PPOI (**a**) and AL (**b**) in cases and controls

Mean hospital stay was 12.1 ± 4.7 days in cases and 16.6 ± 6.8 days in controls, with significance between groups (*F* = 4.164; *t* = 3.494; *p* = 0.001) (Fig. 2). PPOI was significantly more frequent in controls (40, 100%) than in cases (9, 23.7%) (*χ*^2^ = 48.593; *p* < 0.001), and POI mean duration was significantly higher in controls (Table 1; Fig. 3).

AL was evident in 1 patient (2.6%) of cases and 3 patients (7.5%) of controls; AL in 1 patient of cases (LAR group) was treated conservatively with total parenteral nutrition and transanal tube No Coil removed on the 12th day; AL in 3 patients of the control group (2 LAR group; 1 LC group) was treated with loop colostomy. No statistical difference was found in AL occurrence between groups (*χ*^2^ = 0.949; *p* = .327). POI days and AL resulted associated with hospital stay, explaining 45% of the variance (Table 2). POI days were negatively associated with No Coil placement and positively with AL (Table 2). None of the independent variables showed association with PPOI. AL resulted positively associated with POI days, such that an increase of 1 day in POI is associated with 20% increase of AL (log-likelihood-2 = 24.754; Nagelkerke *R*^2^ = .251; Wald = 6.087; OR = 1.229; 95% CI = 1.055–1.599; *p* < 0.014).

**Table 2:** Results of linear regression analysis

Dependent variable	Adjusted *R*^2^	*F*	*p*	Independent variable	*B*	*t*	*p*	95% CI
Hospital stay	0.456	56.061	< 0.001	POI	1.020	6.032	< 0.001	0.683 to 1.357
				AL	6.438	2.538	0.013	1.385 to 11.490
				No Coil	0.013	0.118	0.907	
				Surgery type	0.151	1.785	0.078	
				PPOI	− 0.092	− 0.840	0.404	
POI	0.397	51.734	< 0.001	No Coil	− 3.916	− 7.448	< 0.001	− 5.021 to − 2.902
				AL	5.152	4.275	< 0.001	2.751 to 7.552
				Surgery type	0.116	1.315	0.192	

*Note*: 95% CI are shown only for significant predictors

*POI* postoperative ileus days, *PPOI* prolonged postoperative ileus, *AL* anastomotic leak

## Discussion

Present observational study sought to retrospectively examine the positive effects in terms of postoperative complications and hospital stay of No Coil® implementation in patients undergoing LAR or LC procedures compared to a control group.

Results partly confirm the hypothesis that No Coil placement reduces all postoperative complications.

No Coil resulted in a significant reduction of hospital stay in individuals undergoing both LC and LAR surgeries compared to treatment as usual. To date, this is the first study exploring hospital stay related to No Coil implementation. However, results from linear regression analysis revealed that the most important predictors are longer postoperative ileus days and AL event.

POI days and PPOI, conceivable with gastrointestinal dysmotility occurring during postoperative time, were significantly lower in individuals undergoing LC and LAR with transanal No Coil implementation [11]. Specifically, No Coil placement resulted in a reduction of almost 4 POI days. On the other hand, PPOI events in the control group were extremely higher than expected. In fact, PPOI frequency after colectomy plus defunctioning stoma, or after rectal resection, was previously estimated to be 27% and 30.9%, respectively [12, 30].

In present study, only one patient over 38 of cases experienced AL complication, and the same complication occurred in 3 out of 40 controls. Accordingly, no differences emerged depending on No Coil placement. To the best of our knowledge, only one study explored so far the efficacy of No Coil implementation in reducing AL events. In their study, Montemurro and colleagues examined AL prevalence in a sample of 184 patients undergoing elective total or subtotal proctectomy with low-lying anastomosis and found slightly higher AL estimates (4.8%) compared to present results [13]. Two randomized trial evaluated the use of transanal stent other than No Coil, which are meant to act similarly, although structurally different from it. Amin and colleagues examined the occurrence of AL after LAR plus transanal stent, compared to TAU (defunctioning stoma), and showed anastomotic leakage in three of 41 (about 7%) [18]. Conversely, Bulow and colleagues found that transanal stent was not superior to defunctioning stoma in preventing the risk of AL after LAR (about 10.7%) [19]. Although informative, these studies cannot be compared with present results, given that different devices can result in slightly to moderate differences in efficacy.

AL resulted positively associated with POI days, such that 1 day more of postoperative ileus was associated with 20% increase of AL event.

In light of present results, it can be stated that individuals receiving No Coil placement benefit from lower postoperative ileus days and hospital length of stay with respect to individuals receiving treatment as usual. This is important considering hospital costs are associated with longer hospital length of stay [28]. The most important predictor of AL is POI days, which has been found to be strongly associated with No Coil use [22, 31]. Therefore, it can be hypothesized that No Coil is also contributing to the reduction of AL events, by indirectly reducing POI days. However, these results should be read in light of some limitations.

The sample size was small for both groups and may have prevented to find significance between the two interventions or affected results. The mean age of the sample was around 70 years old; consequently, these results may not be applicable to younger population. Lastly, inter-operator reliability bias cannot be excluded.

However, cases and controls were homogeneous according to gender, age, and type of intervention, excluding these variables as possible confounders and contributing to more consistency. Furthermore, to the best of our knowledge, this is the first study including both LAR and LC types of intervention in the analysis.

These results may preliminarily point out No Coil placement as a valuable procedure assisting colorectal surgery, but further studies are required to confirm and enlarge actual evidence about its efficacy in preventing short- and long-term complications.

## Data Availability

The datasets used and analyzed during the current study are available from the corresponding author on reasonable request
